# The Solution Structure of Heparan Sulfate Differs from That of Heparin: IMPLICATIONS FOR FUNCTION*[Fn FN2]

**DOI:** 10.1074/jbc.M113.492223

**Published:** 2013-08-06

**Authors:** Sanaullah Khan, Ka Wai Fung, Elizabeth Rodriguez, Rima Patel, Jayesh Gor, Barbara Mulloy, Stephen J. Perkins

**Affiliations:** From the ‡ Department of Structural and Molecular Biology, Division of Biosciences, University College London, London WC1E 6BT, United Kingdom and; the § Glycosciences Laboratory, Imperial College London, Department of Medicine, London W12 0NN, United Kingdom

**Keywords:** Analytical Ultracentrifugation, Heparan Sulfate, Heparin, Heparin-binding Protein, Molecular Modeling, X-ray Scattering

## Abstract

The highly sulfated polysaccharides heparin and heparan sulfate (HS) play key roles in the regulation of physiological and pathophysiological processes. Despite its importance, no molecular structures of free HS have been reported up to now. By combining analytical ultracentrifugation, small angle x-ray scattering, and constrained scattering modeling recently used for heparin, we have analyzed the solution structures for eight purified HS fragments dp6–dp24 corresponding to the predominantly unsulfated GlcA-GlcNAc domains of heparan sulfate. Unlike heparin, the sedimentation coefficient *s*_20,_*_w_* of HS dp6–dp24 showed a small rotor speed dependence, where similar *s*_20,_*_w_* values of 0.82–1.26 S (absorbance optics) and 1.05–1.34 S (interference optics) were determined. The corresponding x-ray scattering measurements of HS dp6–dp24 gave radii of gyration *R*_G_ values from 1.03 to 2.82 nm, cross-sectional radii of gyration *R*_XS_ values from 0.31 to 0.65 nm, and maximum lengths *L* from 3.0 to 10.0 nm. These data showed that HS has a longer and more bent structure than heparin. Constrained scattering modeling starting from 5,000 to 12,000 conformationally randomized HS structures gave best fit dp6–dp24 molecular structures that were longer and more bent than their equivalents in heparin. Alternative fits were obtained for HS dp18 and dp24, indicating their higher bending and flexibility. We conclude that HS displays bent conformations that are significantly distinct from that for heparin. The difference is attributed to the different predominant monosaccharide sequence and reduced sulfation of HS, indicating that HS may interact differently with proteins compared with heparin.

## Teaser

**Background:** The polysaccharide heparan sulfate (HS) exhibits key physiological roles.

**Results:** Analytical ultracentrifugation and x-ray scattering revealed extended but bent HS solution structures.

**Conclusion:** Scattering fits resulted in molecular models for HS in solution with glycosidic angles in good accord with a HS crystal structure.

**Significance:** These bent HS models clarify how HS interacts with its ligands.

## Introduction

Heparan sulfate (HS)^2^ is a sulfated glycosaminoglycan that is found extensively on animal cell surfaces and other extracellular surfaces (1, 2). HS has key roles in biological recognition processes at the cell-tissue-organ interface through its interactions with a wide range of proteins (3, 4). Specific interactions involving HS include roles in cell growth and development (5), cell adhesion (6), inflammation and wound healing (7), angiogenesis and cancer (8–10), viral invasion (11, 12), and anticoagulation (13). The breadth of these HS-protein interactions offers potential strategies for therapeutic intervention at the cell-tissue-organ interface.

**FIGURE 1.**
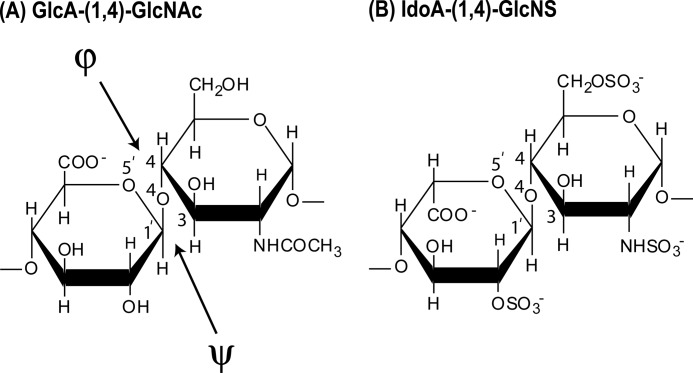
**Chemical structures of the two disaccharide repeats of HS and heparin.** *A*, the major repeating disaccharide unit of HS (glucuronic acid → *N*-acetylglucosamine). The NH·CO·CH_3_ group in the second ring is replaced by NH·SO_3_^−^ in 50% of this structure. The resulting molecular mass of this averaged disaccharide is 483 Da. *B*, the minor repeating unit of HS, which is the major repeating disaccharide unit in 90% of heparin (iduronic acid-2-sulfate → glucosamine-2,6-disulfate). For comparison with this study, heparin is considered to be 50% in the trisulfate form as shown and 50% in a disulfate form where a sulfate group is lost. The resulting molecular mass of this averaged disaccharide is 628 Da.

HS is a sulfated polysaccharide composed of uronic acid and d-glucosamine residue pairs linked by (1→4)-glycosidic bonds (Fig. 1) (14, 15). The uronic acid residue is either unmodified β-d-glucuronic acid (β-GlcA), alternating with *N*-acetylated glucosamine (Fig. 1*A*), or α-l-iduronic acid (α-IdoA), often 2-*O*-sulfated, alternating with *N*-sulfated glucosamine (GlcNS) (Fig. 1*B*). In the latter, sulfation often occurs at C6 and rarely also at C3 (16, 17). HS has a distinct domain organization that is comprised of short *S* domains (IdoA2S and GlcNS residues), long *NA* domains with GlcA and GlcNAc residues, and mixed domain regions at the junctions between the *S* domains and *NA* domains (15, 16). The *S* domains and mixed domain regions are termed the hypervariable regions that result in different functional characteristics for HS from different cell types (16).

Three-dimensional structural studies of HS are required to complete an understanding of the physiological significance of HS-protein interactions. Many structural studies have been carried out for heparin, which is an analog for HS but possesses a higher degree of sulfation, being predominantly *S* region-like in sequence, and for at least 19 heparin-protein co-crystal complexes. This abundance results because of the ease with which heparin is obtained and its strong binding to many of the cell surface proteins whose physiological ligand is HS. An NMR structure is known for heparin (18). Solution structures are known for six purified fragments dp6-dp36 of heparin from constrained scattering modeling; these forms were shown to be similar in conformation to heparin when observed in heparin-protein crystal structures (19). In distinction, up to now, no molecular structures for free HS are known, and only one crystal structure at 0.21-nm resolution for a dp4 HS oligosaccharide complexed with heparinase II is available (20).

Given the importance of understanding the HS solution structure, we have used a multidisciplinary approach to determine molecular structures for HS based on the combination of three methods, namely analytical ultracentrifugation, small angle x-ray scattering, and constrained scattering modeling (21, 22). This approach is well established for solution structure determinations of large multidomain complement and antibody proteins and was recently applied to small heparin oligosaccharide fragments (19, 23). Here, we apply this approach for the second time for oligosaccharide solution structures, this time for eight HS fragments ranging in sizes from dp6 to dp24, thus permitting detailed comparisons with heparin. The HS fragments exhibited solution structures that were distinct from those of the heparin fragments. In particular, their overall lengths are longer compared with heparin, and their structures display a greater degree of bending with increase in size compared with heparin. Our results are attributed to the difference in monosaccharide sequence between HS and heparin fragments, combined with a much reduced degree of sulfation in the HS fragments, which possessed greater structural flexibility than heparin. These results provided new insight on the potential binding modes of HS to proteins.

Following publication of our original 2011 study, we regrettably discovered an error in the anomeric configuration of our heparan sulfate structural models. This present study supersedes the 2011 study, which has been withdrawn.

## EXPERIMENTAL PROCEDURES

### 

#### 

##### Purification of HS Fragments

HS oligosaccharide fragments were prepared according to a similar method to that previously used for heparin oligosaccharides (19, 24–26). Exhaustive heparinase digestion was used to minimize the content of fully sulfated sequences. Approximately 100 mg of HS (prepared from a crude glycosaminoglycan mixture, the kind gift of Laboratori Derivati Organici, Italy: a mixture of HS-I and HS-II as described in Ref. 27) was weighed out and dissolved in ∼2 ml of phosphate buffer, pH 7. An aliquot of 200 μl of heparinase I stock solution was added and left to digest at room temperature for at least 2 h, long enough for the reaction to run to completion. The reaction mixture was evaporated to dryness, using a rotary evaporator at 50 °C.

**FIGURE 2.**
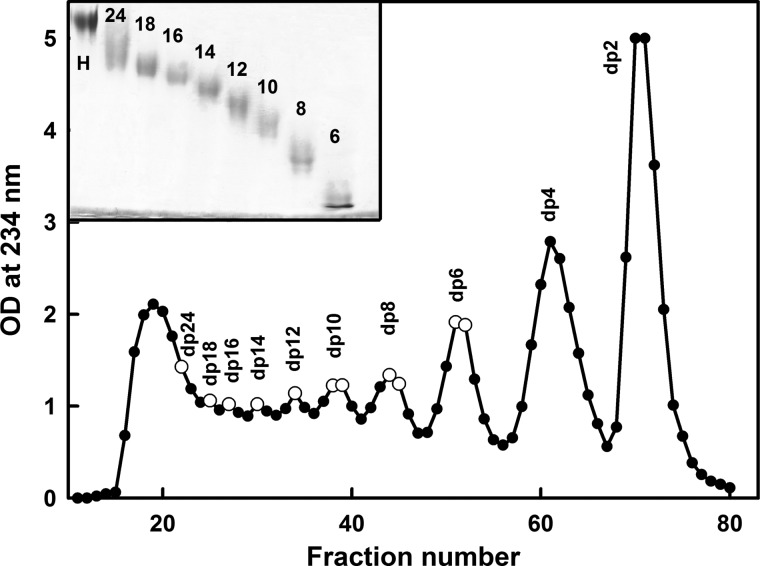
**Purification profile of the HS fragments.** The HS fragments were eluted with a flow rate of 0.2 ml/min using a Biogel P-10 column in 2% ammonium bicarbonate solution. Fractions of 2 ml/10 min were collected, and their HS concentrations were measured spectrophotometrically at 234 nm. The fractions taken for this study are shown by *open circles*. The *inset* shows 25% PAGE of the HS fragments dp6–dp24 (labeled as *6–24*) with heparin dp24 (labeled as *H*) as marker. *OD*, optical density.

To isolate the HS fragments, the dried digest was dissolved in 1.5 ml of 2% ammonium bicarbonate solution and filtered through a 0.45-micron syringe filter before injection onto the preparative gel filtration column. The filtered digested HS was then applied to a preparative gel permeation chromatography column (100 × 1.6 cm; packed with Biogel P10) (Bio-Rad). The HS fragments were eluted using 2% ammonium bicarbonate at a flow rate of 0.2 ml/min in 2-ml fractions. The absorbance of the fractions was measured at 234 nm, and the top fractions corresponding to each individual resolved peak were pooled. The HS oligosaccharides larger than dp12 were not completely resolved (Fig. 2). The pooled fractions were evaporated under reduced pressure and lyophilized before assessment of their sizes by analytical gel permeation chromatography (25). Like heparin, gel permeation chromatography was carried out using two columns (TSK G3000 SW-XL, 30 cm; TSK G2000 SW-XL, 30 cm; Anachem) connected in series. The eluant was 0.1 m ammonium acetate solution at a flow rate of 0.5 ml/min, and HS was detected with a refractive index detector (RI-1530; Jasco). The chromatography system was calibrated using the First International Reference Reagent Low Molecular Weight Heparin for Molecular Weight Calibration (NIBSC 90/686). HS quantization was achieved by integration of the area under each refractive index peak and comparison with a standard curve prepared using known concentrations of low molecular weight heparin. An absorption coefficient of 5500 m^−1^ cm^−1^ at 232 nm was used for HS experiments (28).

##### PAGE of HS Fragments

The HS fragments were analyzed by PAGE to determine the level of purity of each one according to a previously described method (29). Each HS fragment (5 μg) was mixed with 20% glycerol up to a maximum volume of 10–15 μl and then loaded into separate wells (Fig. 2, *inset*). Phenol red in 20% glycerol in a maximum volume of 10 μl was also applied to a separate well as a marker. Initially samples were run through a stacking gel (5% acrylamide, 0.5% bisacrylamide) at 150 V for 20–30 min until the phenol red started to enter the resolving gel. In the resolving gel (25% acrylamide, 1% bisacrylamide), samples were run at a constant current of 18 mA until the phenol red reached the bottom of the gel. The discontinuous buffer system of Laemmli (30) consisted of 0.125 m Tris/HCl, pH 6.8, in the stacking gel and 0.375 m Tris/HCl, pH 8.8, in the resolving gel. The gel running buffer was 25 mm Tris, 0.192 m glycine, pH 8.3. The gel was stained with 0.08% aqueous Azure A for 10 min to visualize HS bands. The gel was then destained in water to remove excess dye and clear the gel background.

##### Analytical Ultracentrifugation of HS Fragments

**FIGURE 3.**
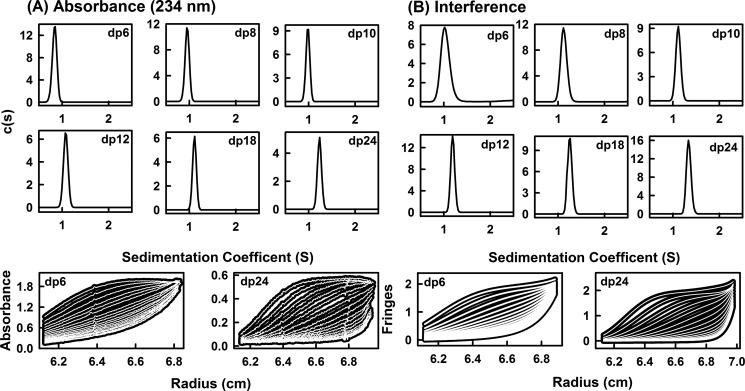
**Sedimentation velocity size distribution analyses *c*(*s*) of six HS dp6–dp24 fragments.** The absorbance and interference boundary scans were fitted using SEDFIT software for the HS fragments, each at 0.5 mg/ml. The mean *s*_20,_*_w_* and their standard deviations are reported in Table 1. *A*, the absorbance data using a wavelength of 234 nm and a rotor speed of 50,000 rpm gave *s*_20,_*_w_* peaks at 0.84 S for dp6, 0.95 S for dp8, 0.98 S for dp10, 1.08 S for dp12, 1.11 S for dp18, and 1.23 S for dp24. Beneath these panels, representative boundary fits are shown for dp6 and dp24, in which only every sixth scan of the 120 fitted boundaries are shown for clarity. *B*, the interference data using a rotor speed of 50,000 rpm gave *s*_20,_*_w_* peaks at 1.04 S for dp6, 1.12 S for dp8, 1.11 S for dp10, 1.19 S for dp12, 1.25 S for dp18, and 1.34 S for dp24. Beneath these panels, representative boundary fits are shown for every sixth scan of the 120 fitted boundaries for dp6 and dp24.

Sedimentation velocity data for eight HS fragments (dp6, dp8, dp10, dp12, dp14, dp16, dp18, and dp24) were obtained on two Beckman XL-I analytical ultracentrifuges (Beckman-Coulter Inc., Palo Alto, CA) using both absorbance and interference optics. Experiments with the dp6–dp24 fragments were performed at concentrations of 0.5 mg/ml in 10 mm HEPES and 137 mm NaCl (pH 7.4). The buffer density was measured at 20 °C using an Anton-Paar DMA5000 density meter to be 1.00480 g/ml. A partial specific volume of 0.467 ml/g determined for heparin (31) was used for HS. An alternative higher value of 0.55 ml/g for HS has been reported elsewhere and was used for data processing only when required to confirm that the partial specific volume has no effect on the outcome of this study (32). Analytical ultracentrifugation runs were carried out in an eight hole AnTi50 rotor with standard double-sector cells with column heights of 12 mm at 20 °C using absorbance optics at 234 nm and interference optics. Sedimentation velocity data were collected at 40,000, 50,000, and 60,000 rpm using absorbance optics and interference optics. The continuous *c*(*s*) analysis method was used to determine the sedimentation coefficients *s*_20,_*_w_* of the eight HS fragments using SEDFIT software (version 9.4) (33, 34). The *c*(*s*) analysis directly fits the experimental sedimentation boundaries using the Lamm equation, the algorithm for which assumes that all species have the same frictional ratio *f*/*f*_o_ in each fit. The final SEDFIT analyses used a fixed resolution of 200, and optimized the *c*(*s*) fit by floating the meniscus and cell bottom when required and holding the *f*/*f*_o_ value, base line, and cell bottom fixed until the overall root mean square deviations and visual appearance of the fits were satisfactory (Fig. 3). The individual *f*/*f*_o_ values calculated previously for the heparin fragments were used for the equivalent HS fragments (19).

##### Synchrotron X-ray Scattering of HS Fragments

X-ray solution scattering of the above eight HS fragments dp6–dp24 were performed on the Beamline ID02 at the European Synchrotron Radiation Facility at Grenoble, France, in two sessions with a ring energy of 6.0 GeV (35). In the first session, data were collected for six HS fragments in 16-bunch mode using beam currents from 63 to 89 mA. In the second session, data were collected for all eight HS fragments in 16-bunch mode using beam currents from 65 to 78 mA. Data were acquired using an improved fiber optically coupled high sensitivity and dynamic range CCD detector (FReLoN) with a smaller beamstop. The sample to detector distance was 3.0 m. Experiments used the same HS concentrations of 0.5 mg/ml and buffers used in the sedimentation velocity experiments. For each HS fragment, the samples were measured in a flow cell, which moved the sample continuously during beam exposure in 10 time frames with different exposure times of 0.1, 0.25, 0.5, and 1 s to check for the absence of radiation damage effects. This exposure was optimized using on-line checks for the absence of radiation damage to show that this was not detectable.

Guinier analyses give the radius of gyration, *R*_G_, which measures the degree of structural elongation in solution if the internal inhomogeneity of scattering within the macromolecules has no effect. Guinier plots at low *Q* values (where *Q* = 4 π sin θ/λ, 2θ is the scattering angle, and λ is the wavelength) gives the *R*_G_ and the forward scattering at zero angle *I*(*0*) (36).

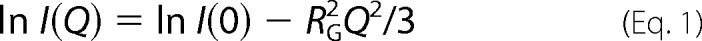
 This expression is valid in a *Q*·*R*_G_ range up to 1.5. If the structure is elongated (*i.e.*, rod-shaped), the radius of gyration of the cross-sectional structure *R*_XS_ and the mean cross-sectional intensity at zero angle [*I*(*Q*)·*Q*]*_Q_*_→0_ parameters are obtained from fits in a higher *Q* range.

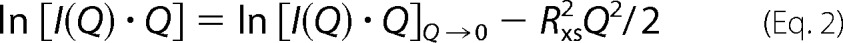
 The *R*_G_ and *R*_XS_ analyses were performed using an interactive PERL script program SCTPL7^3^ on Silicon Graphics OCTANE workstations. Indirect Fourier transformation of the full scattering curve *I*(*Q*) in reciprocal space gives the distance distribution function *P*(*r*) in real space. This yields the maximum dimension of the macromolecule *L* and its most commonly occurring distance vector *M* in real space.

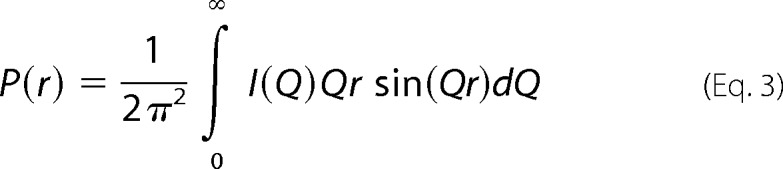
 The transformation was carried out using GNOM software (37). For dp6–dp16, the full x-ray *I*(*Q*) curves contained 295–343 data points in similar *Q* ranges between 0.29 and 1.80 nm^−1^.

##### Molecular Modeling of HS Fragments

Linear HS models were created using the crystal structure of the HS tetrasaccharide (dp4) observed in its complex with heparinase II (PDB code 3E7J) with Discovery Studio (version 2.5) molecular graphics software (Accelrys, San Diego, CA). The monosaccharide residues in the HS tetrasaccharide of 3E7J were N-acetyl α-d-glucosamine (internal), β-d-GlcNAc (reducing terminal), β-d-GlcA (internal), and 4,5-dehydro-d-GlcA (ΔUA; nonreducing terminal). Unfortunately the PDB three-letter abbreviations used in 3E7J do not conform to the PDB conventions. The abbreviation NAG (correctly used only for β-d-GlcNAc) is used for both α- and β-anomers; the abbreviation GCU (correctly used only for α-d-GlcA) is used for β-d-GlcA. In addition, we also point out that the dp4 structure was written out in the original crystallography paper (20) as NAG-GCU-NAG-GCD (where GCD is the PDB code for ΔUA). This order, with the reducing end to the left, is unconventional. In the current study, the disaccharide α-d-GlcNAc-(1→4)-β-d-GlcA, from the two internal monosaccharides in the 3E7J tetrasaccharide, was taken to be the base HS structure, and these disaccharide units (PDB code NDG-BDP) were joined by glycosidic linkages to generate a fully extended linear HS dp30 structure. In this, the phi (Φ) and psi (Ψ) angles were maintained at similar values observed in the starting dp4 structure. Linear HS dp6–dp24 models were created from this extended dp30 starting model by the removal of nonrequired disaccharides.

Totals of 5,000 conformationally randomized models for each of dp6, dp8, dp10 and dp12, 8,000 similar models for each of dp14 and dp16, and 12,000 similar models for each of dp18 and dp24 were created starting from each linear model. In the original HS dp4 structure, the Φ and Ψ angles were −90° and 124° respectively for the GlcA-GlcNAc (BDP-NDG) disaccharide and 85° and 95° respectively for the GlcNAc-GlcA (NDG-BDP) disaccharide. These Φ and Ψ angles were randomized to take any value in a maximum range of ±45° starting from the preceding values using the TorsionKick function in a PERL script that was modified from the ExtractAngle.pl script provided with the Discovery Studio software. For example, in the case of dp16, a total of eight Φ and Ψ angles for GlcA-GlcNAc and seven Φ and Ψ angles for GlcNAc-GlcA were randomized in this way. To avoid steric clashes between the dp16 atoms in each randomized structure, a constant force field termed DREIDING minimization provided in Discovery Studio was used to correct this. DREIDING minimization was useful in generating structures by providing accurate geometries and reasonably accurate steric barrier for organic, biological and inorganic main groups (38).

##### Constrained Scattering and Sedimentation Coefficient Modeling

Each HS model was used to calculate the x-ray scattering curve for comparison with the experimental curve using Debye sphere models (39–41). A cube side length of 0.520 nm in combination with a cutoff of 4 atoms was used to create the spheres for the HS dp6–dp16 models. The hydration shell corresponding to 0.3 g/g H_2_O was created by adding spheres to the unhydrated sphere models using HYPRO (42), where the optimal total of hydrated spheres is listed in Table 1. The x-ray scattering curve *I(Q*) was calculated using the Debye equation as adapted to spheres and assuming a uniform scattering density for the spheres (43). Other details are given elsewhere (39–41, 44). X-ray curves were calculated without instrumental corrections as these were considered to be negligible for the pinhole optics used in synchrotron x-ray instruments. First, the number of spheres *N* in the dry and hydrated models after grid transformation was used to assess steric overlap between the HS disaccharides, where models showing less than 95% of the optimal totals (see Table 1) were discarded. This procedure was found to be insensitive to steric overlap in the case of oligosaccharides, and was discontinued in favor of the DREIDING minimization procedure (above). Next, the models were assessed by calculation of the x-ray *R*_G_ values from Guinier fits of the modeled curves using the same *Q* ranges used for the experimental Guinier fits to allow for any approximations inherent in the use of the *Q*·*R_G_* range up to 1.5. Models that passed the *N* and *R*_G_ filters were then ranked using a goodness-of-fit *R* factor to identify the best fit eight models for each HS fragment.

Sedimentation coefficients *s*^0^_20,_*_w_* for each of the eight best fit HS scattering models were calculated directly from molecular structures using the HYDROPRO shell modeling program (45). The default value of 0.31 nm for the atomic element radius for all atoms was used to represent the hydration shell surrounding HS.

##### Protein Data Bank

The eight best fit dp6–dp24 models are currently available as supplemental materials. They were originally deposited in the Protein Data Bank with accession codes of 3QHG (dp6), 3QHH (dp8), 3QHI (dp10), 3QHJ (dp12), 3QHK (dp14), and 3QHL (dp16). The corrected models have been redeposited with codes of 4KHC (dp6), 4KHD (dp8), 4KHE (dp10), 4KHF (dp12), 4KHG (dp14), 4KHH (dp16), 4KHI (dp18: extended), 4KHJ (dp18: bent), 4KHK (dp24: extended), and 4KHL (dp24: bent).

## RESULTS

### 

#### 

##### Sedimentation Velocity Data Analysis for Eight HS Fragments

The purification profile from the Biogel P-10 column shows that the four smallest HS fragments dp6–dp12 were eluted as well resolved peaks, whereas the four larger fragments dp14–dp18 and dp24 were less well resolved (Fig. 2). Analytical high performance size exclusion chromatography of the HS oligosaccharide fractions as described (25), and PAGE was performed to show that all eight peak fractions showed altered sizes as expected and were relatively homogenous in size. Proton NMR spectroscopy (not shown) showed that the GlcA-GlcNAc disaccharide was the predominant structure present with a minor content of sulfated saccharide residues, presumably originating from transition sequences between the *NA* and *S* domains. Signals typical of heparin-like *S* domains were almost completely absent.

**FIGURE 4.**
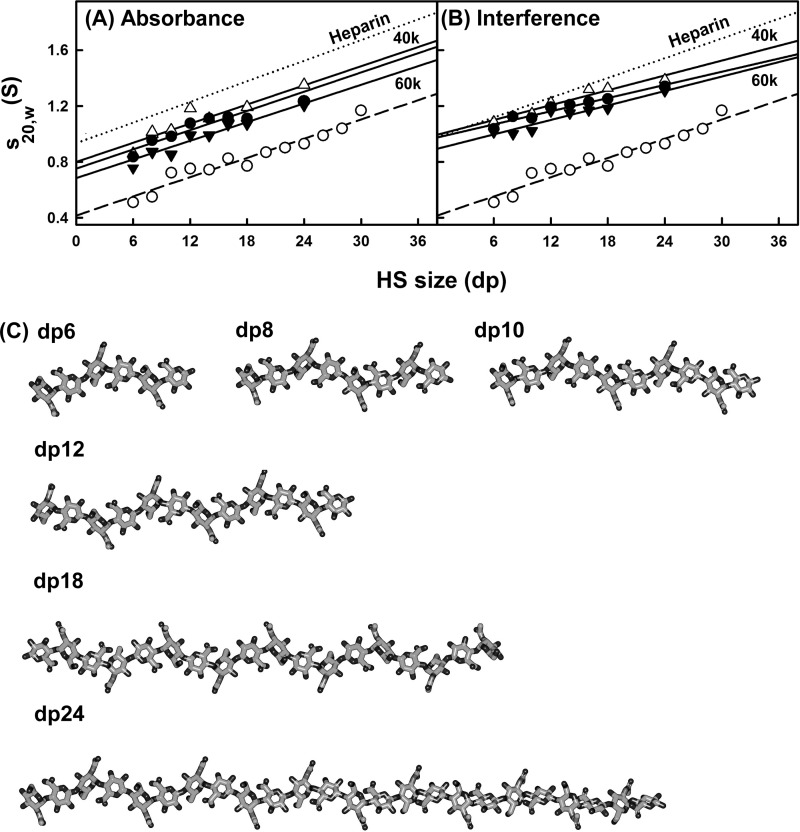
**Comparison of the experimental and predicted sedimentation coefficients for eight HS dp6–dp24 fragments.** The *filled circles* (●) and *triangles* (▴) represent the experimental values for dp6–dp24. The *open circles* (○) represent the predicted values for linear dp6-dp30 models. For comparison, the equivalent heparin experimental data from Ref. 19 is shown as *dotted lines* in *A* and *B. A*, comparison with the experimental sedimentation coefficients at rotor speeds of 40,000 (▵), 50,000 (●), and 60,000 (▾) rpm using absorbance optics. *B*, comparison with the experimental sedimentation coefficients at rotor speeds of 40,000 (▵), 50,000 (●), and 60,000 (▾) rpm using interference optics. *C*, the linear models for HS dp6–dp24 that were created starting from the HS dp4 crystal structure (PDB code 3E7J) are shown.

Analytical ultracentrifugation studies macromolecular structures and sizes through quantitative measurements of sedimentation rates in a high centrifugal field (46). Sedimentation velocity experiments at three rotor speeds were performed for the eight HS fragments (dp6–dp18 and dp24) to determine their shapes and degree of polydispersity. The sedimentation coefficient distribution function *c*(*s*) was calculated by direct fitting of the sedimentation boundaries using SEDFIT software. The absorbance optics analyses for each HS fragment reproducibly resulted in good boundary fits that resulted in single major peaks (Fig. 3*A*). The mean sedimentation coefficient *s*_20,_*_w_* values at three speeds ranged from 0.82 ± 0.05 S for dp6 to 1.26 ± 0.08 S for dp24. The corresponding interference optics analyses for dp6–dp24 also resulted in good boundary fits and single major *c*(*s*) peaks with mean *s*_20,_*_w_* values that ranged from 1.05 ± 0.04 S for dp6 to 1.35 ± 0.04 S for dp24 (Fig. 3*B*). The comparisons in Fig. 4 indicate that the difference between the absorbance and interference *s*_20,_*_w_* values results from statistical variability, and there is no bias between the two data sets. The number of peaks and their widths assess the polydispersity of each HS fragment. In this regard, both the absorbance and interference optics showed better resolution and single narrower major peaks when compared with the equivalent data sets for heparin (Fig. 3 in Ref. 19). The most likely explanation for this appears to be variability in the sulfate content within the heparin fragments, causing variations in mass that resulted in a broader peak width. This effect would not be present in HS because of the reduced sulfation level in HS. The similar single peak widths from either absorbance or interference optics suggest that all eight HS fragments showed narrow size distributions and are relatively homogenous, in agreement with the chromatography results above. The smaller HS fragments show slightly broader peak widths than the larger ones, and this is attributed to a higher back diffusion effect. Unlike heparin, a slight decrease in the *s*_20,_*_w_* values of the absorbance and interference data were observed with increase of rotor speed; this indicated that the *s*_20,_*_w_* values depend on the rotor speed (Fig. 4, *A* and *B*). Inspection of the boundary fits showed that this slight rotor speed dependence resulted from a contribution from back diffusion in the *c*(*s*) fits that was reduced with increase in rotor speed. Accordingly, although the values at the highest speed were taken to be more valid, they were within error of the averaged values (Table 1).

**TABLE 1:** **X-ray scattering and sedimentation coefficient modeling fits for eight HS fragments**

HS fragment	Filter	Number of models	Hydrated spheres*^a^*	*R*_G_ *^b^*	*R*_XS_	*R* factor	Length (*L*)	*s*_20,_*_w_**^c^*
				*nm*	*nm*	%	*nm*	*S*
dp6	None	5,000	8–21	0.71–1.06	0.01–0.44	4.0–9.0	NA	NA
	*R*_G_, *R*_XS_, *R* factor	8	18–19	1.01–1.03	0.30–0.32	4.4–4.6	3.0–3.1	0.47–0.53
	Best fit	1	19	1.02	0.31	4.4	3.1	0.53
	Experimental			1.03 ± 0.08	0.31 ± 0.06		3.0	0.82 ± 0.05
				0.98 ± 0.05				1.05 ± 0.04
dp8	None	5,000	13–28	0.92–1.36	0.10–0.55	4.4–8.3	NA	NA
	*R*_G_, *R*_XS_, *R* factor	8	17–24	1.18–1.20	0.40	4.5	3.5–3.8	0.61–0.62
	Best fit	1	21	1.19	0.40	4.5	3.8	0.61
	Experimental			1.19 ± 0.08	0.40 ± 0.03		3.5	0.94 ± 0.06
				1.16 ± 0.02				1.06 ± 0.08
dp10	None	5,000	16–35	1.03–1.55	0.32–0.68	4.4–11.2	NA	NA
	*R*_G_, *R*_XS_, *R* factor	8	25–29	1.40–1.42	0.43–0.44	4.4	4.5–5.0	0.67–0.68
	Best fit	1	28	1.41	0.44	4.4	4.6	0.67
	Experimental			1.41 ± 0.07	0.44 ± 0.04		4.5	0.95 ± 0.09
				1.37 ± 0.04				1.09 ± 0.06
dp12	None	5,000	19–42	1.10–1.75	0.12–0.78	4.2–12.8	NA	NA
	*R*_G_, *R*_XS_, *R* factor	8	30–40	1.63–1.65	0.48–0.50	4.2–4.5	5.5–5.7	0.74
	Best fit	1	37	1.63	0.5	4.4	5.5	0.74
	Experimental			1.65 ± 0.09	0.49 ± 0.04		5.5	1.08 ± 0.09
				1.62 ± 0.03				1.16 ± 0.05
dp14	None	8,000	21–45	1.20–2.01	0.09–0.81	4.2–11.7	NA	NA
	*R*_G_, *R*_XS_, *R* factor	8	35–39	1.73–1.76	0.48–0.49	4.3	6.0–6.3	0.82–0.83
	Best fit	1	37	1.73	0.49	4.3	6.1	0.82
	Experimental			1.76 ± 0.07	0.51 ± 0.02		6.0	1.07 ± 0.07
				1.82 ± 0.09				1.18 ± 0.04
dp16	None	8,000	27–54	1.22–2.25	0.01–0.93	6.5–16.2	NA	NA
	*R*_G_, *R*_XS_, *R* factor	8	41–48	1.93–2.00	0.49–0.51	6.5–6.6	6.4–7.2	0.86–0.89
	Best fit	1	42	2.0	0.47	6.5	7.2	0.88
	Experimental			2.03 ± 0.07	0.52 ± 0.01		7.0	1.10 ± 0.03
				2.11 ± 0.11				1.24 ± 0.08
dp18	None	12,000	29–60	1.32–2.43	0.01–1.00	5.4–20.2	NA	NA
	*R*_G_, *R*_XS_, *R* factor (extended)	8	45–49	2.24–2.29	0.39–0.48	6.2–7.1	8.0–8.5	0.90–0.92
	Best fit (extended)	1	48	2.25	0.48	6.2	8.5	0.92
	*R* factor (bent)	8	45–53	2.03–2.15	0.38–0.60	5.4–5.6	6.5–7.0	0.92–0.94
	Best fit (bent)	1	46	2.13	0.47	5.4	7.0	0.92
	Experimental			2.34 ± 0.03	0.61 ± 0.05		8.5	1.12 ± 0.06
				2.44 ± 0.11				1.25 ± 0.07
dp24	None	12,000	42–77	1.49–2.85	0.01–1.18	4.6–18.9	NA	NA
	*R*_G_, *R*_XS_, *R* factor (extended)	8	61–64	2.68–2.78	0.52–0.58	6.6–7.9	9.0–10.0	0.91–1.0
	Best fit (extended)	1	61	2.70	0.53	6.6	9.0	0.96
	*R* factor (bent)	8	59–72	2.44–2.59	0.39–0.50	4.6–4.8	8.0–9.0	0.78–1.07
	Best fit (bent)	1	63	2.47	0.57	4.6	8.5	1.07
	Experimental			2.82 ± 0.10	0.65 ± 0.05		10.0	1.26 ± 0.06
				3.0 ± 0.05				1.34 ± 0.06

*^a^* The optimal totals of hydrated spheres were 15 for dp6, 20 for dp8, 25 for dp10, 30 for dp12, 34 for dp14, 39 for dp16, 44 for dp18, and 59 for dp24.

*^b^* The first experimental value is from the Guinier *R*_G_ analyses, and the second one is from the GNOM *P*(*r*) analyses.

*^c^* The averaged experimental *s*_20,_*_w_* value is reported, the first value being from the absorbance (234 nm) data sets and the second value being from the interference data sets. The absorbance and interference data were recorded at rotor speeds of 40,000, 50,000, and 60,000 rpm.

Like heparin, the HS analyses revealed *s*_20,_*_w_* values that increased with an increase in size of the fragments (Fig. 4, *A* and *B*). A typical molecular mass of the most abundant HS dp2 structure shown in Fig. 1*A* is 483 Da, whereas the corresponding value for heparin dp2 is 628 Da (Fig. 1*A* of Ref. 19). Because of the differences in molecular size, the Svedberg equation predicts that the *s*_20,_*_w_* values of the HS fragments will be 77% of those for the equivalently sized heparin fragments. The mean *s*_20,_*_w_* value for HS dp24 is 1.30 ± 0.06 S (Table 1), which is 86 ± 8% of the corresponding value for heparin dp24 of 1.52 ± 0.07 S (Table 1 of Ref. 19). This is almost within error of the mass-predicted reduction in *s*_20,_*_w_* value. If real, the difference between the 77 and 86% values would correspond to a 10% smaller frictional coefficient for HS compared with heparin, *i.e.*, HS may have a slightly more compact solution structure than heparin.

The sedimentation coefficient *s*^0^_20,_*_w_* values were calculated using HYDROPRO software from molecular models of HS. For this, 13 linear HS models (dp6–dp30) were computed starting from the HS dp4 crystal structure seen in its complex with heparinase II (20). Even though the rate of increase of the *s*^0^_20,_*_w_* values with size was predicted correctly, the theoretical *s*^0^_20,_*_w_* values for HS were consistently lower than those seen experimentally (Fig. 4, *A* and *B*). The theoretical values for the HS fragments were consistently lower when compared with their experimental values and therefore show that HS sediments more rapidly than predicted, *i.e.*, the overall solution structures are more compact through bending than the linear HS structures. Below, this difference in Fig. 4 is explained by the best fit bent HS structures that were modeled from the scattering data. It is noteworthy that this difference between experimental and theoretical values was not seen for heparin (19); this shows that the HS and heparin solution structures are different. In addition, the linear HS and heparin structures differ in their degree of elongation. The theoretical *s*^0^_20,_*_w_* value for a linear HS structure is 70% lower than the corresponding theoretical value for a linear heparin structure, in reflection of a longer linear HS structure.

##### X-ray Solution Scattering Data for Eight HS Fragments

**FIGURE 5.**
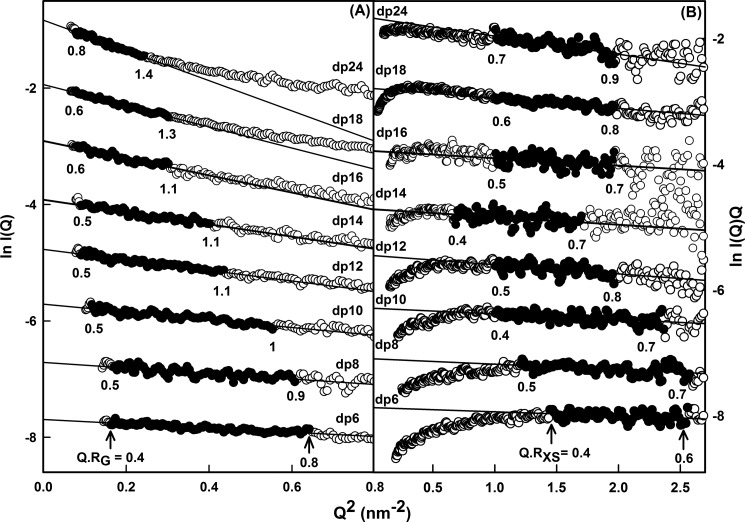
**Experimental Guinier x-ray scattering analyses of eight HS dp6–dp24 fragments.** *A*, Guinier *R*_G_ plots for dp6–dp24 at concentrations of 0.5 mg/ml. The *filled circles* were used to determine the radius of gyration *R*_G_, based on the best fit lines as shown. The *Q* ranges used for the *R*_G_ analyses were 0.40–0.8 nm^−1^ for dp6, 0.42–0.78 nm^−1^ for dp8, 0.34–0.74 nm^−1^ for dp10, 0.30–0.66 nm^−1^ for dp12, 0.30–0.64 nm^−1^ for dp14, 0.29–0.55 nm^−1^ for dp16, 0.28–0.55 nm^−1^ for dp18, and 0.28–0.55 nm^−1^ for dp24. *B*, Guinier *R*_XS_ plots for dp6–dp24. The *filled circles* represent the *Q* ranges used to determine the cross-sectional radius of gyration *R*_XS_, based on the best fit lines as shown. The *Q* ranges used for *R*_XS_ analyses were 1.2–1.6 nm^−1^ for dp6, 1.1–1.6 nm^−1^ for dp8, 1.0–1.54 nm^−1^ for dp10, 1.0–1.44 nm^−1^ for dp12, 0.82–1.3 nm^−1^ for dp14, and 1.0–1.4 nm^−1^ for dp16, dp18, and dp24.

**FIGURE 6.**
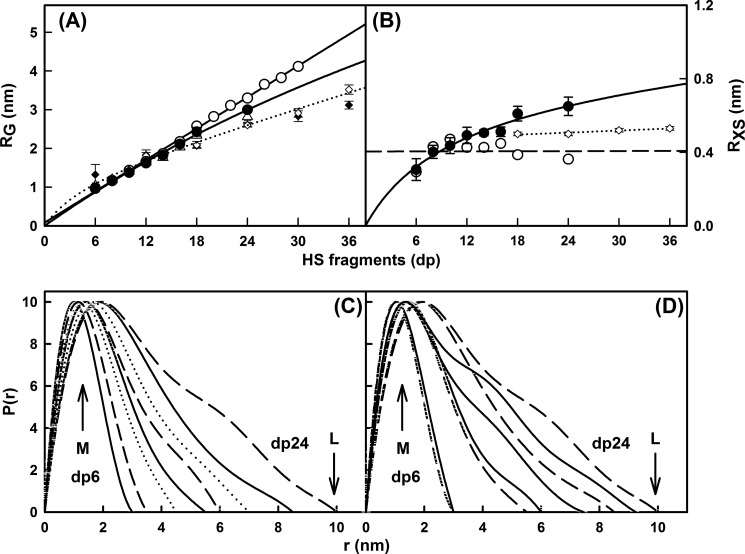
**Experimental Guinier and *P*(*r*) x-ray data analyses of eight HS dp6–dp24 fragments.** *A*, comparison of the experimental *R*_G_ values from Guinier plots (▵) and *P*(*r*) curves (●) with the predicted *R*_G_ values calculated from the linear models of Fig. 4 (○). The six corresponding values for heparin from Ref. 19 are denoted by ♢ and ♦, respectively, and fitted to a *dotted line. B*, comparison of the experimental cross-sectional *R*_XS_ values (●) with the predicted *R*_XS_ values calculated from the linear models of Fig. 4 (○). The corresponding four values for heparin dp18–dp36 from Ref. 19 are denoted by ♢ and fitted to a *dotted line. C*, the distance distribution function *P*(*r*) analyses for dp6–dp24. The *r* values of the maximum at *M* were 1.02 nm (dp6), 1.15 nm (dp8), 1.30 nm (dp10), 1.43 nm (dp12), 1.44 nm (dp14), 1.61 nm (dp16), 1.87 nm (dp18), and 1.90 nm (dp24). The eight fragments are denoted by *continuous*, *dashed*, and *dotted lines* in alternation. *D*, comparison of the *P*(*r*) analyses for HS dp6–dp24 with heparin dp6–dp24. The curves corresponding to the four HS fragments dp6, dp12, dp18, and dp24 are denoted by *dashed lines*, whereas the corresponding four curves for heparin are denoted by *continuous lines*. The heparin *P*(*r*) data are from Ref. 19.

Solution scattering is a diffraction technique that provides structural information on biological macromolecules in random orientations in solution (21, 22). To complement the analytical ultracentrifugation data in more detail, the solution structures of the same eight HS fragments dp6–dp24 at 0.5 mg/ml were characterized by synchrotron x-ray scattering. The scattering experiments reports the scattering curve *I*(*Q*) as a function of scattering angle *Q*. Tests for possible radiation damage effects (“Experimental Procedures”) showed that they were not detectable; accordingly data were acquired with the longest exposure time of 1 s to maximize signal:noise ratios. Guinier analyses of ln *I*(*Q*) *versus Q*^2^ at low *Q* values gives the radius of gyration, *R*_G_, which monitors the degree of macromolecular elongation. Because of the very different sizes of the HS fragments, different *Q* ranges were required for each fragment to work within acceptable linear *Q*·*R*_G_ ranges (Fig. 5*A*). Thus the Guinier fit *Q* range of 0.4 to 0.8 nm^−1^ for dp6 was successively reduced in stages to that of 0.28–0.55 nm^−1^ for dp24 (Fig. 5*A*). The mean Guinier *R*_G_ values increased from 1.03 ± 0.08 nm for dp6 up to 2.82 ± 0.10 nm for dp24 (Table 1). These *R*_G_ values for the eight HS fragments do not show a linear relationship with the size of the HS fragments, unlike the *R*_G_ values calculated from linear models, showing reduced values instead (Fig. 6*A*). Thus the comparison of the *R*_G_ values between the linear models and the experimental data also showed that the HS fragments were bent in solution. In addition, the experimental HS *R*_G_ values are larger for the dp18–dp36 fragments than those seen for the heparin dp18 and dp24 fragments (Fig. 6*A*). This shows that HS has a more elongated structure than that for heparin.

Macromolecules that are sufficiently elongated in shape will show a cross-sectional radius of gyration *R*_XS_ value. The *R*_XS_ value monitors the degree of bending within the macromolecular length. As for the *R*_G_ analyses, different *Q* ranges were used for the *R*_XS_ fits for the different HS fragments depending on the size of the HS fragment, all of which were larger than the *Q* ranges used for the *R*_G_ analyses (Fig. 5*B*). In all cases, despite the worsened signal:noise ratios of the *I*(*Q*) data, linear fit ranges were identified in the plots of ln *I*(*Q*)·*Q versus Q*^2^. These ranges gave experimental *R*_XS_ values of 0.31 nm for dp6 that increased up to 0.65 nm for dp24 (Fig. 6*B* and Table 1). This increase in the *R*_XS_ values correlated with the deviation of the *R*_G_ values from linearity (Fig. 6*A*). They were larger than the calculated *R*_XS_ values of 0.40 nm for the linear HS dp6-dp30 models, thus supporting the conclusion that the HS fragments become progressively more bent with an increase in size. Combination of the *R*_G_ and *R*_XS_ values according to the relationship *L*^2^ = 12 (*R*_G_^2−^ − *R*_XS_^2^) for an elliptical cylinder (36) showed that HS dp6, dp8, dp10, dp12, dp14, dp16, dp18, and dp24 have approximate lengths of 3.4, 3.9, 4.6, 5.5, 5.8, 6.8, 7.8, and 9.5 nm in that order. Similar lengths of 7.0, 9.1, 9.6, and 10.7 nm were observed for the heparin dp18, dp24, dp30, and dp36 fragments (19). In conclusion, the comparison of the dp18 and dp24 lengths showed that HS has a longer structure than that of heparin, in addition to being more bent than heparin.

The distance distribution function *P*(*r*) is calculated from the full *Q* range of the *I*(*Q*) scattering curve (“Experimental Procedures”). The *P*(*r*) curve represents the distribution of all the distances between the atoms within the macromolecule. This provides *R*_G_ values and model-independent determinations of the overall length, *L*, following an assumption of the maximum dimension, *D*_max_ (Fig. 6*C*); note that *L* is not a contour length. The mean *R*_G_ values obtained from the *P*(*r*) curves increase from 0.98 ± 0.05 nm for dp6 to 3.0 ± 0.05 nm for dp24 (Table 1). These *P*(*r*) *R*_G_ values are in excellent accord with the corresponding Guinier *R*_G_ values from the low *Q* values and follow the same trends with size (Table 1 and Fig. 6*A*). Model-independent *L* values are determined from the *r* value where the *P*(*r*) curve reaches zero at large *r*. These experimental *L* values were 3.0 nm for dp6, 3.5 nm for dp8, 4.5 nm for dp10, 5.5 nm for dp12, 6.0 nm for dp14, 7.0 nm for dp16, 8.5 nm for dp18, and 10.0 nm for dp24. These values show increasing deviation with size from the longer lengths measured for the linear HS dp6–dp24 models (*i.e.*, 3.0 nm for dp6, 3.9 nm for dp8, 4.4 nm for dp10, 5.7 nm for dp12, 7.0 nm for dp14, 8.5 nm for dp16, 8.9 nm for dp18, and 11.0 nm for dp24), noting that a hydration shell of thickness 0.6 nm (2 × 0.3 nm) is added to these linear model lengths (44). These experimental *L* values from the *P*(*r*) curves are more accurate compared with the approximate *L* values calculated from the *R*_G_ and *R*_XS_ values that assumed an elliptical cylinder shape for HS; however, these approximate *L* values show that the *R*_G_ and *R*_XS_ values are consistent with the *P*(*r*) analyses. The *P*(*r*) curves also provide the most frequently occurring interatomic distance, *M*, within the heparin structure from the *r* value of the peak maximum. *M* was observed at *r* values that started at 1.02 nm for dp6 and increased to 1.90 nm for dp24 (Fig. 6*C*). In conclusion, these comparisons show that HS has a progressively more bent solution structure with increase in size. Concurrently with this, the lengths, *L*, of HS dp18 and dp24 are longer than those for the corresponding heparin dp18 and dp24 fragments (Fig. 6*D*). Hence, HS is also longer as well as being more bent than heparin.

##### Constrained Modeling of Eight HS Fragments

The experimental x-ray *R*_G_ and *R*_XS_ values showed that the solution structures for HS is longer and more bent than those of heparin. Here, constrained scattering modeling was performed with HS to provide a molecular explanation of these scattering data. The linear models created from the HS dp4 crystal structure were the starting constraint. All eight HS fragments dp6–dp24 were subjected to modeling. They were considered as belonging to a structurally homologous series. As illustrated in the previous modeling of heparin, the linkage connectivity between the oligosaccharide rings was maintained (Fig. 1*A*), whereas the Φ and Ψ rotational angles at each glycosidic linkage was varied randomly in a range of up to ±45° from their preceding values. In all, 5,000–12,000 models for each of the eight HS fragments were generated. For each fragment, x-ray scattering curves were calculated from these randomized models and fitted to the experimental curve. The *R*_G_, *R*_XS_, and *R* factor values were calculated for each modeled curve, where the *R*_G_ and *R*_XS_ values were calculated using the same *Q* range used for the experimental Guinier fits (Fig. 5, *A* and *B*), and the *R* factor is a measurement of goodness of fit. The *R* factor distributions (supplemental Fig. S1, *A–H*) showed that all the dp6–dp24 models (*yellow circles*) encompassed the experimental *R*_G_ values (*dashed lines*) and that the lowest *R* factor values were close to the 5–8% level usually expected for excellent curve fits (47).

**FIGURE 7.**
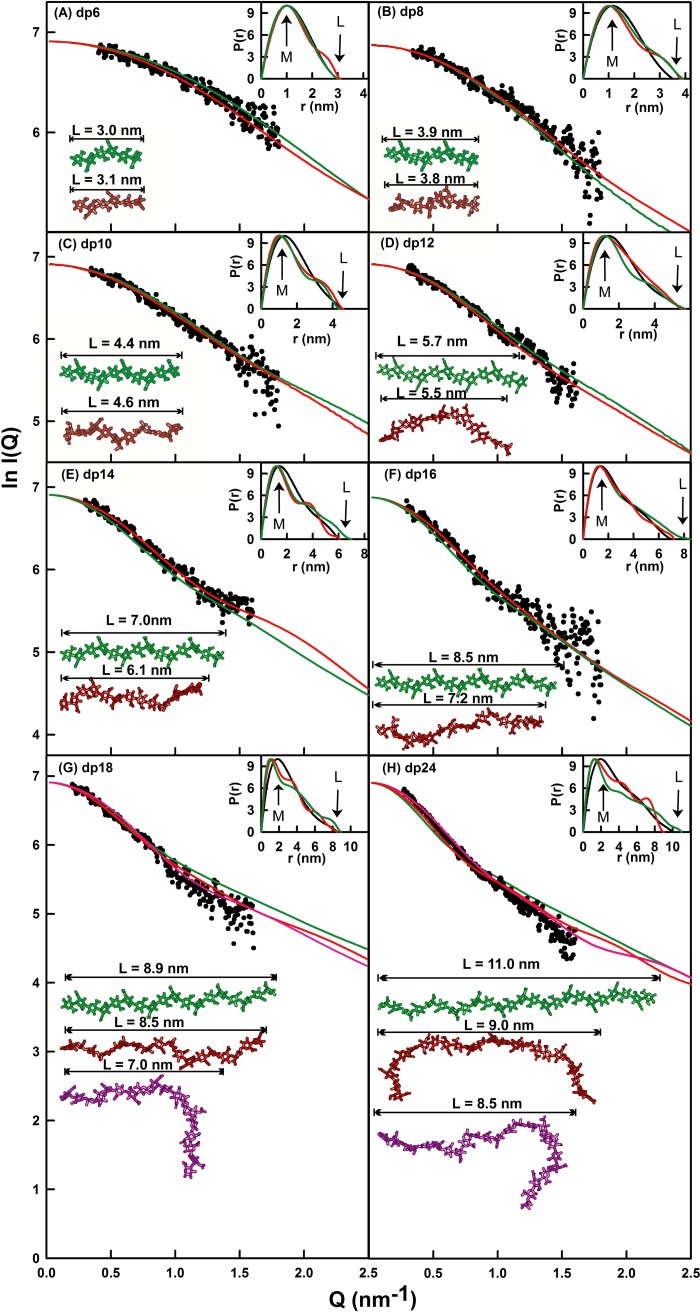
**X-ray modeling curve fits for best fit and poor fit HS dp6–dp24 models.** The main panels (*A–H*) depict the *I*(*Q*) curve fits, and the *insets* show the *P*(*r*) distance distribution function fits. The experimental *I*(*Q*) and *P*(*r*) scattering data are represented by *black circles* or *lines*, respectively; the *red lines* and models correspond to the best fit dp6–dp24 models from the trial and error searches; and the *green lines* and models correspond to the linear poor fit dp6–dp24 models from Fig. 4. The best fit and linear models are shown in the *left lower corner*, together with their maximum lengths *L* in nm for comparison with the experimental *L* values in the *P*(*r*) curves. For dp18 and dp24, the best fit model identified from only the minimum *R* factor value as filter is shown in *purple*.

Totals of 5,000 models for dp6–dp10 and 8,000 models for dp12–dp16 provided enough randomized conformers to be able to determine best fit HS solution structures. Typically the lowest *R* factors showed the best agreement with the experimental x-ray curves. Near these minima, the best fit *R*_G_ values for dp6–dp16 (*one red* and *seven cyan circles*) agreed well with the experimental *R*_G_ values. The best fit *R*_XS_ values for dp6–dp16 also showed good agreement with the experimental *R*_XS_ values (supplemental Fig. S1, *I–P*). In distinction, the linear models for the dp6–dp16 HS fragments showed greater deviations from the experimental *R*_G_ and *R*_XS_ values (*green circles*). These modeling analyses (Table 1 and Fig. 7) confirmed that a nearly linear structure with slight bending accounted for the x-ray and analytical ultracentrifugation data for dp6–dp16.

For HS dp6, the eight best fit models gave *R* factors of 4.4–4.6%, *R*_G_ values of 1.01–1.03 nm, *R*_XS_ values of 0.30–0.32 nm, and maximum lengths, *L*, of 3.0–3.1 nm. These agree well with the experimental *R*_G_ value of 1.03 ± 0.08 nm, *R*_XS_ value of 0.31 ± 0.06 nm, and the *P*(*r*) length of 3.0 nm (Table 1). In distinction to these agreements, the linear dp6 model gave an *R* factor of 4.1%, an *R*_G_ value of 0.98 nm, and a *L* value of 3.0 nm. The visual agreement between the experimental and modeled *I*(*Q*) curves and *P*(*r*) curves was excellent (Fig. 7*A*). The calculated *s*^0^_20,_*_w_* values from the eight best fit models gave 0.47–0.53 S. These values are lower but comparable with the experimental *s*_20,_*_w_* values of 0.82 ± 0.05 and 1.05 ± 0.04 S, given that the typical accuracy of the *s*^0^_20,_*_w_* calculation is ± 0.21 S (22).

For HS dp8, the modeling analyses indicated slightly bent structures similar to those seen for dp6. The eight best fit models gave *R* factors of 4.5%, *R*_G_ values of 1.18–1.20 nm, *R*_XS_ values of 0.40 nm, and *L* values of 3.5–3.8 nm. These values agree well with the experimental *R*_G_ value of 1.19 ± 0.08 nm, *R*_XS_ value of 0.40 ± 0.03 nm, and the *P*(*r*) length of 3.5 nm (Table 1). In distinction to these agreements, the linear dp8 model again showed a higher *R* factor of 4.8% and an *R*_G_ value of 1.21 nm, an *R*_XS_ value of 0.43 nm, and a larger *L* value of 3.9 nm. The visual agreement of the experimental and modeled *I*(*Q*) and *P*(*r*) curves was excellent (Fig. 7*B*). The eight calculated *s*^0^_20,_*_w_* values of 0.61–0.62 S are smaller but comparable with the experimental *s*_20,_*_w_* values of 0.94 ± 0.06 and 1.06 ± 0.08 S.

For HS dp10, the modeling analyses showed good agreements with slightly bent structures, in which the deviation from a linear dp10 structure for dp10 was slightly increased. The eight best fit dp10 models gave *R* factors of 44%, *R*_G_ values of 1.40–1.42 nm, *R*_XS_ values of 0.43–0.44 nm, and *L* values of 4.5–5.0 nm. These correspond well with the experimental *R*_G_ value of 1.41 ± 0.07 nm, *R*_XS_ value of 0.44 ± 0.04 nm, and *L* value of 4.5 nm (Table 1). The deviations from a linear dp10 model are larger, for which the *R* factor is 4.4%, the *R*_G_ value is 1.44 nm, the *R*_XS_ value is 0.47 nm, and the *L* value is 4.4 nm. The visual agreement of the experimental and modeled *I*(*Q*) and *P*(*r*) curves was again excellent (Fig. 7*C*). The eight modeled *s*^0^_20,_*_w_* values of 0.67–0.68 S are again comparable with the experimental *s*_20,_*_w_* values of 0.95 ± 0.09 and 1.09 ± 0.06 S.

For HS dp12, the modeling analyses were also successful, in which the deviation from a linear dp12 structure was greater. The eight best fit models gave *R* factors of 4.2–4.5%, *R*_G_ values of 1.63–1.65 nm, *R*_XS_ values of 0.48–0.50 nm, and *L* values of 5.5–5.7 nm. These agree well with the experimental *R*_G_ value of 1.65 ± 0.09 nm, the *R*_XS_ value of 0.49 ± 0.04 nm, and the *L* value of 5.5 nm (Table 1). The linear dp12 model showed a higher *R* factor of 4.5%, a higher *R*_G_ value of 1.68 nm, a reduced *R*_XS_ value of 0.42 nm, and a longer *L* value of 5.7 nm. The visual agreement of the experimental and modeled *I*(*Q*) and *P*(*r*) curves was excellent (Fig. 7*D*). The eight modeled *s*^0^_20,_*_w_* values of 0.74 S are comparable with the experimental *s*_20,_*_w_* values of 1.08 ± 0.09 and 1.16 ± 0.05 S.

For HS dp14, good agreements between the models and experimental data were obtained, whereas the deviation from a linear dp14 structure was larger. The eight best fit dp14 models gave *R* factors of 4.3%, *R*_G_ values of 1.73–1.76 nm, *R*_XS_ values of 0.48–0.49 nm, and *L* values of 6.0–6.3 nm. These values agree well with the experimental *R*_G_ value of 1.76 ± 0.07 nm, the *R*_XS_ value of 0.51 ± 0.02 nm, and the *L* value of 6.0 nm (Table 1). These values deviated from the linear dp14 model, which had an *R* factor of 4.7%, a higher *R*_G_ value of 1.91 nm, a lower *R*_XS_ value of 0.38 nm, and an *L* value of 7.0 nm. Again the visual agreement of the experimental and modeled *I*(*Q*) and *P*(*r*) curves was excellent (Fig. 7*E*). The eight modeled *s*^0^_20,_*_w_* values of 0.82–0.83 S compare well with the experimental *s*_20,_*_w_* value of 1.07 ± 0.07 and 1.18 ± 0.04 S, respectively.

For HS dp16, the outcome of the modeling analyses was similar to that of dp14. The eight best fit models gave *R* factors of 6.5–6.6%, *R*_G_ values of 1.93–2.00 nm, *R*_XS_ values of 0.49–0.51 nm, and *L* values of 6.4–7.2 nm. These agree well with the experimental *R*_G_ value of 2.03 ± 0.07 nm, the *R*_XS_ value of 0.52 ± 0.01 nm, and the *L* value of 7.0 nm (Table 1). The linear dp16 model gave a poorer fit with an *R* factor of 6.7%, an *R*_G_ value of 2.15 nm, an *R*_XS_ value of 0.38 nm, and an *L* value of 8.5 nm. The experimental and modeled *I*(*Q*) and *P*(*r*) curves showed excellent agreement (Fig. 7*F*). The eight modeled *s*^0^_20,_*_w_* values of 0.86–0.89 S are comparable with the experimental *s*_20,_*_w_* values of 1.10 ± 0.03 and 1.24 ± 0.08 S.

Of interest were the different outcomes seen with HS dp18 or dp24, starting from 12,000 randomized models for these two HS structures. Following the procedures used for dp6–dp16, the best fit extended models for dp18 and dp24 gave reasonable *R* factors of 6.2–7.9%. For extended dp18, modeled *R*_G_ values of 2.24–2.29 nm, *R*_XS_ values of 0.39–0.48 nm, and *L* values of 8.0–8.5 nm were obtained. Unlike dp6–dp16, the modeled *R*_G_ and *R*_XS_ values were less than the experimental *R*_G_ value of 2.34 ± 0.03 nm and the *R*_XS_ value of 0.61 ± 0.05 nm (Table 1). As expected, the linear model gave a poorer fit with an *R* factor of 8.2%, *R*_G_ value of 2.22 nm, an *R*_XS_ value of 0.31 nm, and an *L* value of 8.9 nm. For extended dp24, modeled *R*_G_ values of 2.68–2.78 nm, *R*_XS_ values of 0.52–0.58 nm, and *L* values of 9.0–10.0 nm were obtained. The modeled *R*_G_ and *R*_XS_ values were also less than the experimental *R*_G_ value of 2.82 ± 0.10 nm and the *R*_XS_ value of 0.65 ± 0.05 nm (Table 1). The linear model also gave a poorer fit with an *R* factor of 11.9%, an *R*_G_ value of 2.72 nm, an *R*_XS_ value of 0.43 nm, and an *L* value of 11.0 nm. Notably, the *R*_G_ values of 2.13 nm (dp18) and 2.47 nm (dp24) at the *R* factor minima of supplemental Fig. S1 (*G* and *H*) (Table 1) did not coincide with the experimental *R*_G_ values of 2.34 nm (dp18) and 2.82 nm (dp24). Better agreements were observed for more bent models (Table 1). These differences in *R*_G_ values suggested that conformational heterogeneity between extended and bent structures was present, *i.e.*, dp18 and dp24 exhibited multiple conformations in solution. The experimental and modeled *I*(*Q*) and *P*(*r*) curves showed good visual agreements for either conformation (Fig. 7, *G* and *H*). For dp18, the modeled *s*^0^_20,_*_w_* values of 0.90–0.92 S (extended) or 0.90–0.94 S (bent) were similar to the experimental *s*_20,_*_w_* values of 1.12 ± 0.06 and 1.25 ± 0.07 S, with the best fit models giving two values of 0.92 S. For dp24, the modeled *s*^0^_20,_*_w_* values of 0.91–1.0 S (extended) or 0.78–1.07 S (bent) were similar to the experimental *s*_20,_*_w_* values of 1.26 ± 0.06 S and 1.34 ± 0.06 S, with the two best fit models giving a value of 0.96 or 1.07 S.

## DISCUSSION

The size and spacing of *S* domains in HS are proposed to be as important to its biologically significant interactions with proteins as are the detailed sequences of the *S* domains themselves (49). Heparin, a commonly used model compound for HS, consists of lengthy *S* domains, made up largely of the repeating trisulfated disaccharide shown in Fig. 1*B*, separated by much smaller, unsulfated *NA* domains. In HS the position is reversed, and long *NA* domain sequences (Fig. 1*A*) act as spacers to separate the short *S* domains. Although the *S* domain conformation, exemplified by heparin, has been the subject of numerous studies (50), the *NA* domain has not. It has been proposed that the *NA* domain repeating sequences are both less flexible (51) and more flexible (52) than the *S* domains.

The application of constrained scattering modeling has proved to be as effective for the HS fragments as it was for heparin previously. Usually scattering fits are performed for protein structures of size 20–100 kDa and higher (21, 22). The HS fragments dp6–dp24 and the heparin fragments dp6–dp36 are notably smaller in size with masses of 1–7 and 2–11 kDa, respectively. The ability to measure their scattering curves was attributed to the high x-ray beam intensity and low backgrounds at the instrument, together with improved detector technology. Constrained scattering modeling determines a three-dimensional molecular structure that best accounts for the observed scattering curve through trial and error searches that rule out structures that are incompatible with the observed scattering curves. By fixing the analyses to what is already known about the macromolecule, namely the carbohydrate rings, and varying only the Φ and Ψ angles of each glycosidic linker, relatively few modeling variables are involved in the scattering fits. Through the variation of Φ and Ψ, the resulting 5,000–12,000 models provided sufficient statistical detail to result in clear V-shaped graphs of *R* factor *versus R*_G_ and *R* factor *versus R*_XS_ values. The best fit models were identified by the lowest *R* factors, and they were verified by the agreement of the modeled and experimental *R*_G_ and *R*_XS_ values at this point. The quality of the HS dp6–dp16 scattering fits was similar to those of the heparin dp18–dp36 fits (19). Interestingly, different fits were obtained for the HS dp18 and dp24 structures. The monodispersity of these two fragments had been established by the single peaks seen in the ultracentrifugation *c*(*s*) analyses (Fig. 3); thus the potential contribution of sample heterogeneity in dp18 and dp24 can be ruled out as an explanation for the different fits. The ability to fit either extended or bent HS dp18 and dp24 structures to the scattering curves resulted in the conclusion of multiple conformations for HS dp18 and dp24. This modeling outcome is distinct from that for heparin dp6–dp36, where only single conformations were required for good fits. This outcome suggested that the heparin structures show greater rigidity than the HS structures.

The HS fragments used in this study were produced by extensive depolymerization using heparinase 1, an enzyme that cleaves only within the *S* domains (28) leaving *NA* domains untouched. It is therefore likely that some minor degree of sulfation remains at the reducing and nonreducing end of our fragments, but that internal disaccharides are unsulfated. Such fragments bear a closer resemblance to *NA* domains of intact HS than the most commonly used model compound for this sequence, the capsular polysaccharide from *Escherichia coli* K5 (51, 52). These structures provide novel comparative insight into the structures of HS and heparin, and the likely manner that these two polyanionic oligosaccharides interact with their protein ligands. HS and heparin both share similar covalent structures (Fig. 1). The comparison of our two sets of structures for HS and heparin becomes essentially that between *NA* and *S* domains. The greater bending and flexibility of HS compared with heparin (see Fig. 9) may be attributed to the difference in uronic acid residue, in which GlcNAc alternates with GlcA in HS, causing HS to adopt a distinct conformation from that of fully sulfated heparin (53).

**FIGURE 8.**
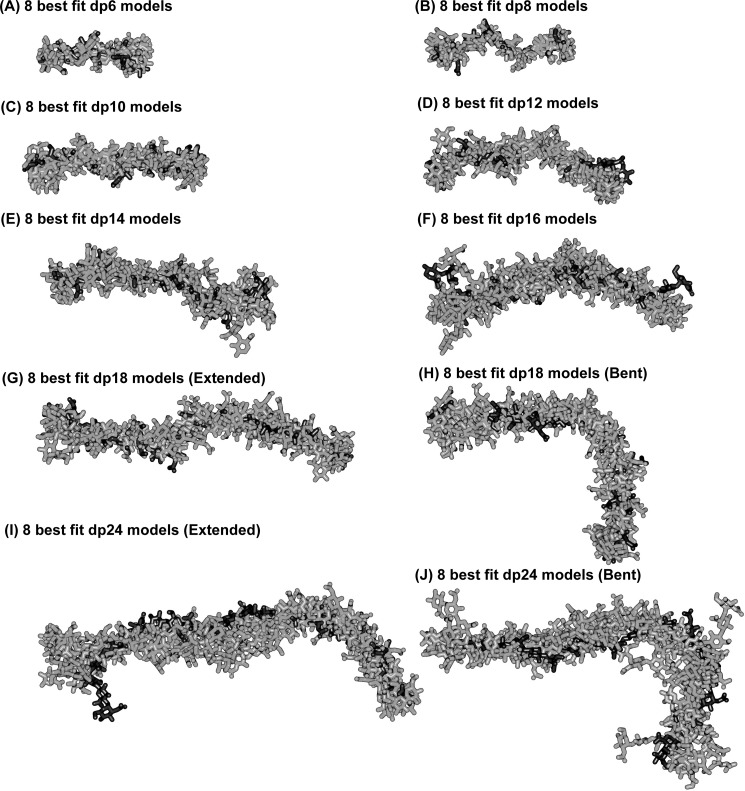
**Superimposition of the eight best fit models for each of the eight HS dp6–dp24 fragments.** Each set of eight best fit models for the eight HS fragments were superimposed globally using Discovery Studio VISUALISER software, and their non-hydrogen atoms are displayed as shown. Each best fit model from Fig. 7 is shown in *black*, whereas the seven related best fit structures are shown in *gray*. For dp18 and dp24, both the overall extended best fit structures (filtering on *R*_G_, *R*_XS_, and *R* factor values) and the bent best fit structures (filtering on *R* factor values only) are shown.

**FIGURE 9.**
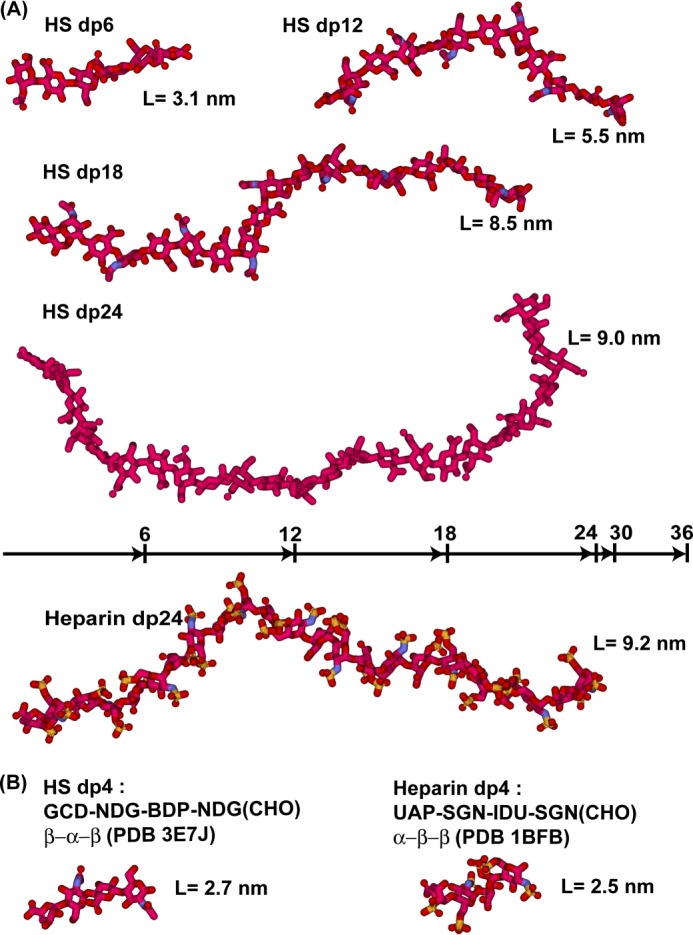
**Comparison of the best fit HS dp6–dp24 structures with the equivalent heparin dp6–dp24 structures.** *Red*, carbon and oxygen; *blue*, nitrogen; *yellow*, sulfur. *A*, four best fit HS models (dp6, dp12, dp18 (extended), and dp24 (extended)) are compared with the heparin dp24 model at the bottom all drawn to the same scale. The lengths of heparin dp6, dp12, dp18, and dp24 are indicated above the heparin dp24 structure for comparison with HS. *B*, the glycosidic linkages in the crystal structure of HS dp4 complexed with heparinase II (PDB code 3E7J) are compared with the crystal structure of the complex of heparin dp4 with fibroblast growth factor (PDB code 1BFB). The anomeric configurations of the glycosidic linkers are shown as α or β.

More detailed inspection of the best fit models for HS dp6–dp16 clarifies their progressively more bent structures in solution with increase in HS size. This outcome is visible from the superimposition of the eight best fit models for each HS fragment (Fig. 8). When comparing the solution structures of heparin dp18 and dp24 (Fig. 8 of Ref. 19) with those of HS dp18 and dp24, the HS structures were visibly more bent than those of heparin (Fig. 9*A*). Crystal structures containing HS or heparin showed that the glycosidic linkage in HS has a similar length to that in heparin (Fig. 9*B*). In HS, the separation between the C1–C4 atoms of GlcA-GlcNAc is 0.237 ± 0.003 nm and that between GlcNAc-GlcA is 0.235 ± 0.002 nm. For heparin, analyses of five crystal structures containing dp6 showed that very similar separations were seen between IdoA-GlcNS of 0.241 ± 0.004 nm and between GlcNS-IdoA of 0.243 ± 0.006 nm. Thus the increased length of HS in solution compared with heparin is mostly the consequence of altered Φ and Ψ angles. In terms of rotational bend, it is already known that the crystallographic Φ and Ψ angles for smaller HS and heparin structures agree with each other within error (Table 2 and supplemental Fig. S2). The Φ and Ψ angles for HS dp6–dp24 were all similar, including those in the small crystal structures (Table 2). The linkage geometries of our HS models fall approximately into the low energy regions for α(1→4) and β(1→4) linkages between glucose residues, as determined for maltose and cellobiose by experimental and theoretical methods (48). Thus the mean Φ and Ψ angles for the GlcA-GlcNAc linkage were −85 and 127° in HS, being similar but not identical with the corresponding IdoA-GlcNS values of −61 and 132° in heparin. The mean Φ and Ψ angles for the GlcNAc-GlcA linkage were 85 and 91° in HS, close to the corresponding values of 98 and 86° for GlcNS-IdoA in heparin (Table 2). The distribution of the Φ and Ψ values in supplemental Fig. S2 (*A* and *B*) showed low rotational variability at both glycosidic linkages in HS. These distributions were in good accord with those for heparin in supplemental Fig. S2 (*C* and *D*). In conclusion, the more bent structure in solution for HS than for heparin (Fig. 9*A*) is attributed to small but reproducible differences of 24 and 13° for the two Φ angles of heparin and HS (Table 2). In contrast, the two Ψ angles of heparin and HS differ by only 5°.

**TABLE 2:**
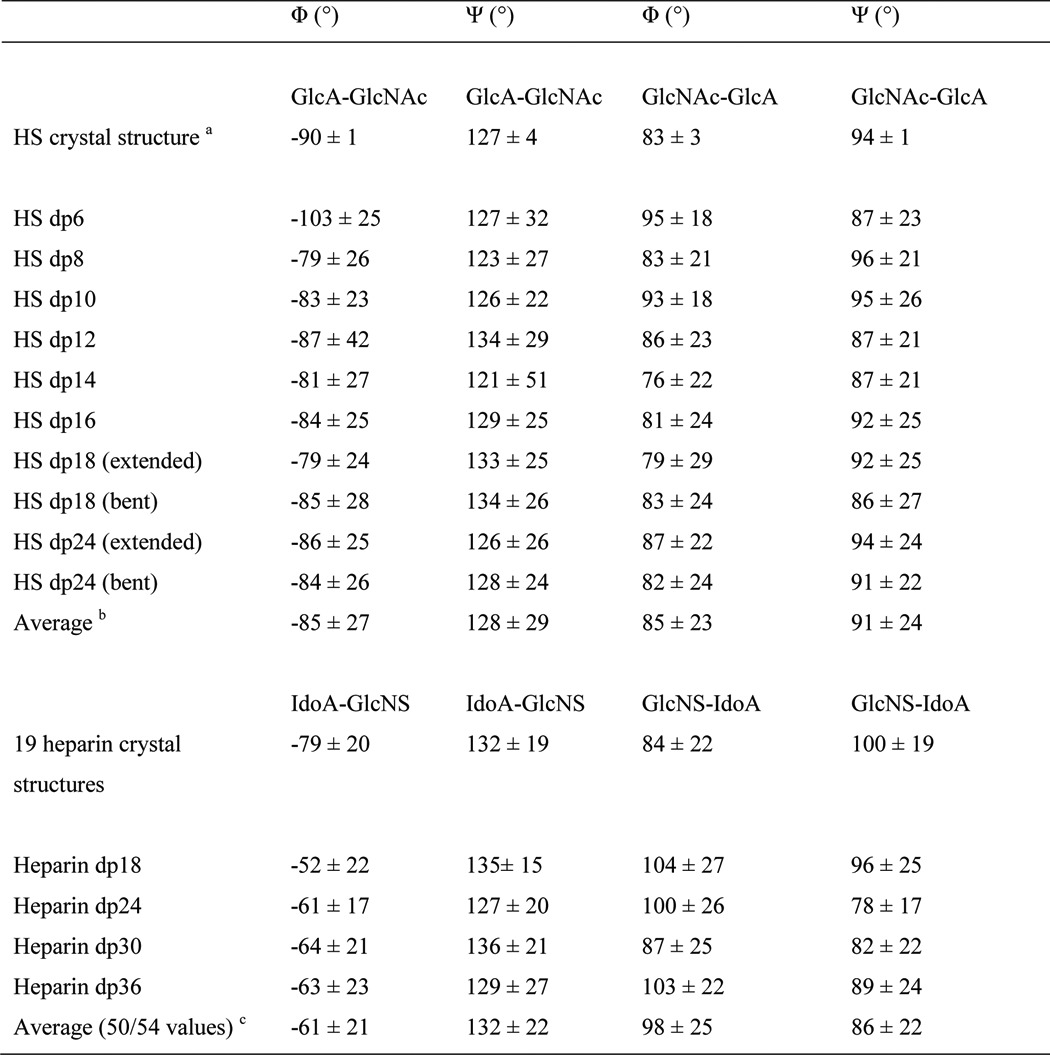
**Summary of the Φ and Ψ angles in the crystal and solution structures of HS and heparin**

*^a^* The mean value from two HS dp4 molecules seen in the crystal structure (PDB code 3E7J).

*^b^* The average is calculated from all the Φ and Ψ angles in the eight best fit models.

*^c^* The average is calculated from the heparin models of Ref. 19.

The solution structures of the HS dp6–dp24 fragments exhibited bending and flexibility (Figs. 8 and 9*A*). In addition, the HS structures are slightly longer for reason of alterations in the glycosidic Φ and Ψ angles (supplemental Fig. S2). The physical basis for these changes in HS is likely to arise from the GlcA-GlcNAc sequences (as opposed to the IdoA-GlcNS sequences in heparin). Unlike HS, heparin will be influenced by greater repulsion between regular repeats of sulfate-sulfate, sulfate-carboxylate, and carboxylate-carboxylate groups. The combination of the *NA* and *S* domain structures within the parent HS structure suggests that different parent HS structures with greater or lesser bending may arise through variations of the ratio in sizes of the *NA* and *S* domains. These variations are likely to be as important as the fine structure of the individual domains in the physiological functions of these complex glycosaminoglycans (49, 55).

In terms of biological function, the greater degree of bending in HS accounts for the ability of HS to bind to a diverse range of protein ligands in all orientations. This will facilitate the assembly of large multipartner complexes on cell surfaces such as those involving the 20-domain structure of complement factor H (56). The outcome of 19 protein-heparin crystal structures has been discussed previously (19), whereas only one protein-HS crystal structure is known (20). It was of interest that the Φ and Ψ angles for the HS crystal structure are similar to those seen in the 19 heparin-protein crystal structures (Table 2). In addition, the mean Φ and Ψ angles for HS in solution are similar and close to those seen by crystallography for HS dp4. Several studies of HS-protein interaction have noted that SAS sequences, in which two short S domains are separated by an *NA* sequence, are preferred for optimum binding (48). This is particularly true for oligomeric proteins such as, for example, MIP-1a or platelet factor 4 (7, 57). In these cases, the multiple heparin-binding sites on the oligomer are not always arranged in a linear way, so that a single long S domain cannot readily bind to more than one site on the same multimer. It has been reasonable to suppose that *NA* domains, composed of alternating α(1→4)- and β(1→4)-linked hexopyranoses, would be more flexible than the unusually stiff heparin structure of *S* domains, allowing an SAS domain to bend to present two S domains to heparin-binding sites on opposite sides of a protein complex. The conclusion from our present study of HS *NA* fragments supports this intuitive reasoning.

## Footnotes

3 J. T. Eaton and S. J. Perkins, unpublished software.

## Footnotes

2 The abbreviations used are:

HS: heparan sulfate
dp: degree of polymerization
GlcA: d-glucuronic acid
IdoA: l-iduronic acid
GlcNS: *N*-sulfated glucosamine
PDB: Protein Data Bank.

## Addendum

In our originally reported HS best fit structures (58), the anomeric configurations of the GlcA and GlcNAc residues should have alternated between α and β. Instead, the anomeric configurations were all β in our original HS models. The present study replaces our original study (58), which has now been withdrawn. The error was traced back to a misunderstanding of the starting HS structure in the Protein Data Bank, and we take full responsibility for our mistake. First, the dp4 structure had been written out in the original crystallography paper (20) as NAG-GCU-NAG-GCD (where NAG is the PDB code for GlcNAc, GCU is the code for GlcA, and GCD is the code for ΔUA). This order, with the reducing end to the left, is unconventional and was a contributing factor to our misunderstanding. Second, the use of the terms NAG and GCU corresponded to the β-GlcNAc and α-GlcA anomers, respectively, in the PDB dictionary. In fact, HS is comprised of the α-GlcNAc and β-GlcA anomers and should have been described as NDG and BDP in the HS dp4 crystal structure (PDB code 3E7J). Inspection of PDB depositions containing GCU showed the existence of other terminological confusions. Several relatively recent structures in the PDB use GCU for internal β-GlcA residues, possibly following the example of the classic hyaluronate and chondroitin sulfate studies (PDB codes 1HYA, 2HYA, 4HYA, 1C4S, and 2C4S). In addition to the PDB code 3E7J of the present study, the structures of bee venom hyaluronidase with a hyaluronate tetrasaccharide (PDB code 1FCV) and CD44 with a hyaluronate octamer (PDB code 2JCQ) use GCU and not BDP. The PDB structure 2JCR (from the same study as PDB code 2JCQ) uses the correct term BDP. Related discrepancies in the PDB have also been reported by others (54, 59).
